# Speech Analysis Using Artificial Intelligence as a Peri-Operative Evaluation: A Case Report of a Patient with Temporal Lobe Epilepsy Secondary to Tuberous Sclerosis Complex Who Underwent Epilepsy Surgery

**DOI:** 10.3390/brainsci11050568

**Published:** 2021-04-29

**Authors:** Keiko Niimi, Ayataka Fujimoto, Yoshinobu Kano, Yoshiro Otsuki, Hideo Enoki, Tohru Okanishi

**Affiliations:** 1 Department of Rehabilitation, Seirei Hamamatsu General Hospital, 2-12-12 Sumiyoshi, Nakaku, Hamamatsu, Shizuoka 430-8558, Japan; k-niimi@sis.seirei.or.jp; 2 Comprehensive Epilepsy Center, Seirei Hamamatsu General Hospital, 2-12-12 Sumiyoshi, Nakaku, Hamamatsu, Shizuoka 430-8558, Japan; enokih.neuropediatr@gmail.com (H.E.); t.okanishi@tottori-u.ac.jp (T.O.); 3 Seirei Christopher University, 3453 Mikatagahara, Kitaku, Hamamatsu, Shizuoka 433-8558, Japan; 4 Faculty of Informatics, Shizuoka University, 3-5-1 Johoku, Nakaku, Hamamatsu, Shizuoka 430-8011, Japan; kano@inf.shizuoka.ac.jp; 5 Department of Pathology, Seirei Hamamatsu General Hospital, 2-12-12 Sumiyoshi, Nakaku, Hamamatsu, Shizuoka 430-8558, Japan; otsuki@sis.seirei.or.jp

**Keywords:** speech analysis, epilepsy, artificial intelligence, intelligence quotient, surgery

## Abstract

Background: Improved conversational fluency is sometimes identified postoperatively in patients with epilepsy, but improvements can be difficult to assess using tests such as the intelligence quotient (IQ) test. Evaluation of pre- and postoperative differences might be considered subjective at present because of the lack of objective criteria. Artificial intelligence (AI) could possibly be used to make the evaluations more objective. The aim of this case report is thus to analyze the speech of a young female patient with epilepsy before and after surgery. Method: The speech of a nine-year-old girl with epilepsy secondary to tuberous sclerosis complex is recorded during interviews one month before and two months after surgery. The recorded speech is then manually transcribed and annotated, and subsequently automatically analyzed using AI software. IQ testing is also conducted on both occasions. The patient remains seizure-free for at least 13 months postoperatively. Results: There are decreases in total interview time and subjective case markers per second, whereas there are increases in morphemes and objective case markers per second. Postoperatively, IQ scores improve, except for the Perceptual Reasoning Index. Conclusions: AI analysis is able to identify differences in speech before and after epilepsy surgery upon an epilepsy patient with tuberous sclerosis complex.

## 1. Introduction

Improvements in cognition [1], mood [2] and social skills [3] have been reported after epilepsy surgery. Such differences have been explained hypothetically by improvements in the intelligence quotient (IQ) after epilepsy surgery [4,5]. In the real world, the fact that not all patients who undergo epilepsy surgery show postoperative improvements in IQ has also been reported [6,7]. However, caregivers and physicians sometimes notice unexplained postoperative improvements in daily activities.

Human beings are highly sensitive to detecting subtle differences in characteristics such as vocal tone, intonation, pause, pitch, speed and accent in conversation [8,9]. Hence, why differences in conversational ability are often recognized postoperatively, though such recognition is merely subjective, as opposed to meeting objective criteria.

In other words, even for individuals with the same IQ, characteristics such as the form of speech, intonation and vocal tone change due to changes in their underlying condition, for instance, with regard to concentration, emotional ups and downs and fatigue.

The subtle sensing of change is currently considered subjective evaluation, but the application of artificial intelligence (AI) methodologies to identify differences in speech before and after surgery may result in the characterization of objective criteria to explain previously subjective observations.

A patient with temporal lobe epilepsy secondary to tuberous sclerosis complex (TSC) undergoing epilepsy surgery is presented. We hypothesize that AI analysis will allow the identification of differences in speech before and after surgery. With the aim of devising a method to objectively analyze whether conversation is more fluent postoperatively, a preliminary analysis of this patient with epilepsy before and after epilepsy surgery is, therefore, conducted using AI.

## 2. Method

Written, informed consent for the publication of this case report was obtained from the patient’s guardian. The ethics committee at Seirei Hamamatsu General Hospital approved the protocol for this study (approval no. 3287).

**Figure 1:**
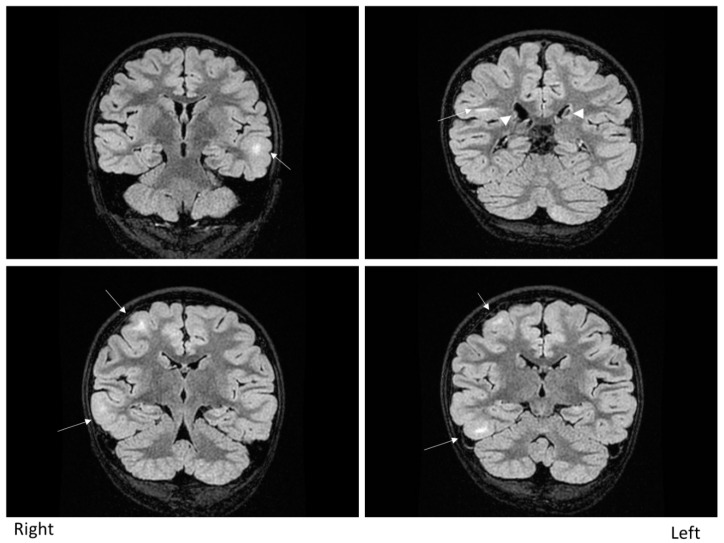
Brain magnetic resonance imaging (MRI). Fluid-attenuated inversion-recovery (FLAIR) MRI shows multiple cortical and subcortical tubers (arrows). Subependymal nodules are indicated by arrows.

The patient was a nine-year-old girl with epilepsy secondary to TSC. She started experiencing focal-onset impaired awareness seizures from six months of age. The seizure frequency was weekly. Brain magnetic resonance imaging showed multiple cortical and subcortical tubers (Figure 1). Electroencephalography (EEG) showed frequent epileptiform discharges over bilateral hemispheres independently, comprising multiple independent spike foci. Ictal events captured by long-term video-EEG showed stereotypical seizures arising from the right frontotemporal area. The patient, therefore, underwent invasive monitoring followed by a right anterior temporal lobectomy with amygdalohippocampectomy. In addition to epilepsy secondary to TSC, this patient also had juvenile facial angiofibroma, hypopigmented macules (“ash leaf” patches) on the limbs, shagreen patches on the back and polycystic kidneys with no angiomyolipomas. No abnormalities were apparent on cardiological, dental and ophthalmological studies. Genetically, a *TSC2* missense mutation was confirmed.

Both one month before and two months after the surgery, IQ testing and speech analysis were conducted. For the IQ testing, the fourth edition of the Wechsler Intelligence Scale for Children (WISC-IV) was used [10,11]. For the speech analysis, the patient was asked to look at and explain four frames of a cartoon from the Standard Language Test of Aphasia, developed by the Japanese Society of Higher Brain Dysfunction [12]. The four frames of the cartoon consisted of the following scenes: (1) a man wearing a hat and walking with a cane; (2) the man’s hat is suddenly blown off by the wind; (3) the man chases after his hat, which is blown into a pond; (4) the man uses his cane to retrieve his hat from the pond.

The patient’s verbal explanations of these scenes were recorded, manually transcribed and annotated and then automatically analyzed using AI software [13]. This AI software performs classification tasks by machine learning; it was originally trained to classify psychiatric diseases such as depression, anxiety and dementia using more than 300 h of recorded conversations between subjects and doctors. In a previous study, we performed a feature sensitivity analysis to identify training features that are effective, based on support vector machine (SVM) classifications. Since we did not have a sufficient number of recorded samples in the present case to train the machine learning model for this specific task, the AI software was used without any additional training to extract training features for automatic analysis, and the features from the previous study were used as they were, even though an AI model is first calibrated using training data and its generalizability is examined using test data that play no role in the calibration [14]. The automatic analysis included audio features such as volume, formant (frequency), speed and linguistic features such as lexical statistics and parts of speech (e.g., nouns, verbs, adjectives) (Table 1). Our linguistic analysis was performed using a linguistic parser that outputs features such as word segments, word base forms, parts of speech and surface cases. The core part of this parser was implemented using a conditional random field model, which is a type of machine learning model used for sequential tagging. The parser was trained by newspaper texts with gold-standard tags annotated by humans. We used a customized dictionary for this parser to obtain a better performance for speech.

The patient remained seizure-free for at least 13 months after radical treatment. Postoperatively, her WISC-IV scores improved, except for the Perceptual Reasoning Index.

## 3. Results

Table 1 shows the detailed results of the analyses. It is well known that the word frequency per lexicon is effective for classifying mental states. Content words, which exclude functional words such as particles and prepositions from the entire lexicon, are also an important feature, more important than simply measuring the total number of uttered words. Particles are the other effective clue in the Japanese language as they show complex linguistic structures, which are difficult to generate when linguistic ability decreases. In this case, in terms of speech analysis, the total numbers of morphemes and objective case markers (“*wo*” in Japanese) per second increased, whereas the number of subjective case markers (“*ga*” in Japanese) per second decreased. In the Japanese language, there are explicit case markers for each case role, which appear as particles, e.g., “*ga*” is a subjective case marker particle, “*wo*” is an objective case marker particle, etc. For example, “the man is picking up his hat” was translated into Japanese as “*sono* (the) *otokonohito* (man) *ga* (subjective case marker) *boushi* (his hat) *wo* (objective case marker) *hirottsuteiru* (is picking up)”. In the acoustic analysis, total utterance time was longer preoperatively than postoperatively, even though she performed the same task.

The neuropathology of the resected anterior temporal lobe showed large, cytoplasm-rich, grotesque cells, mainly in the white matter, accompanied by sporadic gliosis and balloon cells.

## 4. Discussion

AI analysis showed differences in speech between before and after surgery in a patient with temporal lobe epilepsy secondary to TSC. The results from this case indicated that the patient incorporated large amounts of information into her conversation within a short time postoperatively. In the present case, both speech and WISC-IV scores increased postoperatively [4], so the results of speech analysis could be considered to have improved.

One of the differences detected by AI analysis between the preoperative and postoperative speech was vocal pitch (frequency). The human voice is said to be related to emotional intelligence [15]. Humans can sense the emotional states of others based on characteristics such as vocal tone, volume and frequency, and communicate with each other depending on emotional states [16,17]. What the AI analysis detected in this patient might reflect some degree of change in emotional intelligence. Another difference detected by the AI analysis was an increased number of morphemes per second. This was probably because of improved IQ; the patient might have started to manage more language information than before surgery. AI analysis also detected a decrease in the number of subjective case markers and an increase in the number of objective case markers per second. Subject and object verbs are chosen and expressed based on the psychological state, such as fear, fright, hate and delight [18]. This might be related to the fact that the number of subjective case markers per second decreased, whereas the number of objective case markers per second increased. Such changes in case markers also reflect higher cognitive abilities.

In this study, AI analysis at least identified differences in speech before and after surgery. However, the size of the training data is always a problem; it is difficult, especially in the clinical domain, to prepare a large amount of data. Researchers in the computer science domain are tackling the small data issue by selecting an appropriate machine learning method and suitable number of parameters to make the training converge. Recent machine learning methods, like Transformer and its variants (for example, BERT, Lent, etc.), require huge numbers of training data as these models need to train their huge numbers (>billion) of training parameters. Few-shot and zero-shot learning, which are recent trends in the AI area, could be options as they require only tens or a couple of data samples.

SVM, which does not require such large numbers of training samples, was used to avoid this issue in our previous study [13], and it showed an accuracy of approximately 80% for classifying each mental disease compared to healthy people, even though we built the world’s largest database of recorded conversations between subjects (more than 300 h). These accuracy scores were obtained by cross-fold validation to obtain stable evaluations. This pretrained model was applied in the present study.

For accurate AI analysis, data from a greater number of participants need to be collected, and both preoperative and postoperative factors need to be compared statistically. However, since it would be difficult to obtain huge sample numbers of patients with a rare disease, it would be necessary to study whether AI obtained from related diseases could be applied when dealing with rare diseases.

## 5. Conclusions

AI analysis was able to discriminate differences in speech before and after epilepsy surgery upon an epilepsy patient with TSC.

## Figures and Tables

**Table 1:** Results of speech analysis using artificial intelligence and intelligence quotient testing.

		Pre-Surgery	Post-Surgery
Linguistic Analysis	Morphemes/s	3.3	3.6
	Content words/s	1.4	1.6
	Vocabulary/s	1.4	2.4
	Lexicon of content words/s	0.8	1.1
	Nouns/s	0.3	0.5
	Adjectives/s	0.05	0.00
	Verbs/s	0.30	0.50
	Adverbs/s	0.09	0.10
	Objective case markers/s	0.05	0.10
	Subjective case markers/s	0.14	0.10
	Postpositional particles/s	0.36	0.80
Acoustic Analysis	Total utterance time (s)	22	10
	Mean frequency (Hz)	603.9	863.9
	Maximum volume (dB)	0.776	0.824
	Mean volume (dB)	0.060	0.200
IQ results	Full IQ	66	73
	VCI	74	76
	PRI	68	66
	WMI	73	88
	PSI	73	88

IQ, intelligence quotient; VCI, verbal comprehension index; PRI, perceptual reasoning index; WMI, working memory index; PSI, processing speed index.

## Data Availability

The data are not publicly available due to patients’ privacy.
